# Implementation of an Evidence-Based Depression Care Management Program (PEARLS): Perspectives From Staff and Former Clients

**Published:** 2012-04-26

**Authors:** Lesley Steinman, Margaret Cristofalo, Mark Snowden

**Affiliations:** University of Washington Health Promotion Research Center; University of Washington, Seattle, Washington; University of Washington, Seattle, Washington

## Abstract

**Introduction:**

Although researchers develop evidence-based programs for public health practice, rates of adoption and implementation are often low. This qualitative study aimed to better understand implementation of the Program to Encourage Active, Rewarding Lives for Seniors (PEARLS), a depression care management program at a Seattle-King County area agency on aging.

**Methods:**

We used stratified, purposive sampling in 2008 to identify 38 PEARLS clients and agency staff for participation. In 9 focus groups and 1 one-on-one interview, we asked participants to identify benefits and negative consequences of PEARLS, facilitators of and barriers to program implementation, and strategies for overcoming the barriers. Two independent researchers used thematic analysis to categorize data into key themes and subthemes.

**Results:**

PEARLS benefits clients by decreasing depression symptoms and addressing other concerns, such as health problems. For staff, PEARLS provides "another set of eyes" and is a comprehensive program to help them meet clients' mental health needs. Barriers included issues with implementation process (eg, lack of communication) and the perception that eligibility criteria were more rigid than those of other agency programs. Recommended solutions included changing eligibility criteria, providing additional staff training, increasing communication, and clarifying referral procedures, roles, and responsibilities.

**Conclusion:**

Barriers to PEARLS delivery discourage referrals to what is generally viewed as a beneficial program. Implementing participants' strategies for overcoming these barriers can enhance delivery of PEARLS to a greater number of older adults and help them improve their depression symptoms.

## Introduction

Although researchers develop evidence-based programs for public health practice, rates of adoption and implementation are often low (1), and incorporating knowledge from randomized controlled trials into routine practice takes an average of 17 years (2-4). A challenge in translation research is to adequately understand the context in which the intervention will be implemented (5,6). "Context" may include organizational components and other external influences, such as staff attitudes and knowledge, availability of funding, policies and regulations, and organizational culture and climate (1,7).

The Program to Encourage Active, Rewarding Lives for Seniors (PEARLS) is a home-based, care management program to detect and manage depression among community-based older adults (www.pearlsprogram.org). PEARLS was developed in partnership with community-based aging organizations and designed for delivery outside of traditional mental health settings. Two randomized controlled trials (8-10) demonstrated that PEARLS resulted in significantly lower severity and greater remission of late-life depression.

The primary objective of this study was to better understand the context in which PEARLS was implemented at a community-based organization. We aimed to ascertain the benefits and negative consequences of PEARLS for clients, staff, the agency, and others (eg, family members, health care providers); facilitators of and barriers to program implementation; and strategies for improving PEARLS implementation.

## Methods

### Design

A qualitative research design is well-suited for understanding context (11). We used focus group interviews to collect data between March and September of 2008 in Seattle, Washington, from homogeneous groups of people involved in PEARLS implementation (12,13); focus groups are advantageous because participants consider their views in relation to those of others (14,15). The research was approved by the University of Washington's institutional review board.

### Study setting

Since the original PEARLS effectiveness study was completed, the local area agency on aging (Aging and Disability Services [ADS]) has continued to implement PEARLS. Most ADS clients are low-income, frail elders who are homebound and socially isolated. Although ADS has some components for successfully implementing evidence-based programs (6,7), its ability to adequately reach its target population and fully adopt PEARLS (16) has been limited. Recent ADS client data (2007-2010) suggest that only 10% of depressed elders have been referred to PEARLS, compared with more than one-third of eligible ADS clients referred during the randomized controlled trial 2000-2003) (unpublished ADS data, 2005).

### Sampling and recruitment

We used stratified, purposive sampling (17) in 2008 to identify ADS administrators and supervisors, ADS case managers (who make PEARLS referrals), PEARLS counselors, and former PEARLS clients. For staff, inclusion criteria included current employment at ADS in one of these roles. Former PEARLS clients were eligible if they had completed PEARLS during the past year. Our goal was to recruit all PEARLS program staff (2 PEARLS counselors and 3 program administrators), at least 15 ADS case managers and supervisory staff (of 58 total), and 3 former clients (of 15 total). We recruited staff via flyers, e-mails, and word-of-mouth and former clients by telephone via case managers or PEARLS counselors. We enrolled 9 program administrators, 18 case managers, 4 supervisory staff, and 7 PEARLS clients. No participants dropped out after enrollment.

### Data collection

The research team developed a focus group guide. Each topic was introduced by a question, followed by several probes. A research interviewer trained in semistructured interviewing and focus group techniques conducted 10 interviews (9 focus groups and 1 one-on-one interview) (Table). Focus groups (range, 2-7 participants) were uniform in role; that is, case managers were grouped with other case managers. One individual interview was conducted with the PEARLS clinical supervisor; this person has a distinct role and other staff members often defer to this person, a situation that could affect what is shared in a group setting. The final sample size (N = 38) was determined by theoretical saturation (when new data no longer brought additional insights to the research questions). Interviews lasted 45 minutes to 2 hours and were audio taped and transcribed verbatim. Participants gave written informed consent at the start of each group.

### Data analysis

We used thematic analysis, a research method to analyze text data with attention to the content or contextual meaning (18). Using this method, a researcher examines language for the purpose of classifying large amounts of text into categories that represent similar meanings (19). This method provides accounts of participants' experiences with PEARLS implementation and identifies the broader contextual issues that shaped their experiences.

Two members of the research team (M.C. and L.S.) analyzed and coded the data. They used transcripts to independently identify subthemes in each major theme (eg, program benefits), then met to compare their coding. Several passages used to identify each subtheme were compared to provide evidence for thematic selections. When disagreements arose, coders presented supporting textual passages and reasoning until a final subtheme list was developed. Coders met with study participants and other ADS staff to confirm whether interpretations and findings were appropriate; after this comparison, no results needed to be changed. No statistical software was used to analyze the data.

## Results

### Benefits of PEARLS

**For clients**

In addition to treating depression, PEARLS was credited with other benefits, such as increasing clients' physical and pleasurable activities, social support, quality of life, function, physical health, and ability to live independently, all of which improve clients' depression symptoms and are benefits unto themselves. As a PEARLS staff member (focus group no. 5) shared,

I think the treatment really does improve their depression. Once that happens, or at least along with that happening, I think people become more activated; they tend to take better charge of their medical care and their physical health, in addition to being less depressed. They tend to manage things in their lives a bit better.

Participants cited beneficial program components. Its home-based setting was discussed as being essential in reaching frail, homebound older adults who are hindered by transportation barriers and stigmas, particularly about accessing traditional mental health care. Certain program processes were also viewed favorably. As a former client (focus group no. 9) shared,

That form [the PEARLS worksheet, which includes the 7 problem-solving steps and action plans for physical and social activity and pleasant events] was really beneficial too, [be]cause it helped me focus on certain issues instead of just being so scatter-brain[ed] and not knowing what to do with it all. And having something on paper helped, as I had something to refer to.

One case manager (focus group no. 3) articulated that these components became tools that "can be applied to the other stresses that come up in clients' lives."

**For staff**

PEARLS counselors provide support to the case manager and the goals of case management, and case managers and other agency staff benefited from having someone else in the client's home to provide feedback on the client's status and to assist with other concerns. As a case manager (focus group no. 7) described,

When the [PEARLS counselor] goes out to see people, that is another set of eyes and ears with the client that can see things that aren't quite right. . . . He can convey to us the client's needs and concerns.

PEARLS also offers a more in-depth service than the triage and referral that typically defines case management. As a case manager stated (focus group no. 4), PEARLS "touches the client directly," providing a more "comprehensive" and "proactive" tool that case managers can offer.

**For agency**

Participants indicated that implementing PEARLS has been innovative for ADS because area agencies on aging typically do not provide mental health services and that delivering PEARLS helps the agency feel they are doing more about depression for their clients. ADS has also "garnered a lot of respect from the national case management community" (case manager, focus group no. 6), serving as a model for policy making and as a source of pride. Through PEARLS, ADS has gained experience with evidence-based programming by partnering with the University of Washington and now serves new populations with novel programming. Finally, PEARLS provided ADS new funding opportunities, such as a county health and human services levy to offer PEARLS to veterans and their spouses.

**Others outside the agency**

Participants indicated that PEARLS clients' family, friends, and neighbors, as well as providers of other health, mental health, and social service systems, benefit from clients being less depressed and having enhanced skills to manage their lives. According to a PEARLS counselor (focus group no. 8), the program has the potential to save or offset care costs because of decreased use of other services:

If people are caring for their depression as well as their health, it would certainly help the cost of the health care. They will be using services more appropriately and maybe they will also not let conditions get so severe that they are an emergency.

### Negative consequences from implementing PEARLS

Few negative consequences of the PEARLS program were identified by clients; some focus group participants reported no negative consequences. Negative consequences included clients being disappointed if determined to be ineligible for the program and stress or embarrassment because of the stigma of mental illness. As a PEARLS team member (focus group no. 2) described, "Sooner or later people have to accept that they're depressed, and that does put them face-to-face with stigma about being mentally ill." Participants also indicated that challenges may arise when the client drops out (eg, feeling of failure on the part of the client) or finishes the program (eg, clients having difficulty transitioning out of intervention, feeling of being abandoned).

Negative consequences for staff and the agency were framed as barriers to program implementation.

### Facilitators for implementing PEARLS

Participants spoke of processes that facilitated program delivery. Counselors aid implementation through advertising PEARLS in the agency by using personalized flyers, presentations at staff meetings, and individual contacts with case managers. Counselors also provide tools for case managers to connect clients to PEARLS. As a case manager (focus group no. 6) stated,

What I like is the picture [of the PEARLS counselor] . . . and the little advertisement flyer about the program because I take those with me just like I do the forms. And when I'm doing the assessment and the client already scored that mark of 5 or better, I already have that something to hand to the client. So it's a flyer that's used as an advertising tool as well as a door-opening.

### Barriers to implementing PEARLS

Program exclusion criteria were the most commonly identified barriers to program implementation. The following exclusion criteria, in order of importance, were mentioned: speaking a non-English language, having too few depressive symptoms or too many (ie, major depression), having comorbid psychiatric conditions (eg, schizophrenia), having substance abuse disorders, being younger, and having cognitive impairment. As a case manager (focus group no. 6) said, "It seemed to me that there was more screening out than screening in." These program eligibility criteria were viewed as too stringent in the context of an increasingly younger and sicker client population, coupled with frequent pressure to refer to PEARLS. The criteria also present a discrimination issue, conflicting with the agency's goal to serve all clients, particularly those who are non-English–speaking, who comprise one-third of the agency's client population and are served by other programs through contracts with interpreters.

These eligibility criteria dissuaded case managers and supervisors from referring clients to PEARLS, despite their beliefs in the many benefits of the program. As a case manager (focus group no. 7) described,

Sometimes it is kind of a hopeless feeling, like, gosh, they are going to be screened out right away . . . we feel like we've got this option, and we would love to connect you up with the program, but we can't because you're not going to be eligible.

Another widespread barrier for delivering PEARLS was the depression screening instrument. PEARLS actively screens for depression, which is often unrecognized in older people. Potential clients are first screened using the 11-item Center for Epidemiologic Studies Depression Scale (CES-D-11) (20,21). Clients are then screened for minor depression with the 9-item Patient Health Questionnaire (PHQ-9) (22) when referred to PEARLS. Some staff were frustrated at having to administer 2 screenings, given the many assessments they already perform. Other participants perceived the CES-D-11 as flawed, expressing the belief that it does not accurately identify some clients' depressive symptoms, is culturally inappropriate, does not distinguish depressive symptoms from other medical conditions, and is not answered truthfully by clients who fear the stigma of mental illness.

Workload was another barrier to program implementation. Case managers who have high caseloads indicated that referring clients to PEARLS can become burdensome. One case manager (focus group no. 7) said,

There is more expected of the case managers for each client, so sometimes when you see someone who is depressed and you are required to offer the person a referral, you think, "I've been with this client almost 2 hours already . . . I'm not even halfway through the assessment, and I have got to be back in the office in an hour." And I don't feel you have the opportunity to present the program to the client.

### Strategies for improving PEARLS implementation

Focus group participants identified possible strategies for addressing barriers to program delivery and enhancing program benefits and facilitators. Changing the eligibility criteria was the most common approach mentioned. As a case manager (focus group no. 4) stated, "Boy, if you opened up English-as-a-second-language clients, I probably have 3 clients I could refer right now." Another strategy was marketing PEARLS outside of the agency, as many other providers (eg, physicians, formal caregivers) see agency clients more regularly, presenting more opportunities to refer.

Improving communication was mentioned by participants in every interview. Better communication and feedback (eg, information on how the referral process works) is needed between case managers and counselors. More communication is also needed about roles and responsibilities for implementation, such as who is responsible for following up on the outcome of the PEARLS referral. Participants also asked for more frequent program updates from administrators to staff members to better institutionalize PEARLS into regular agency practice.

## Discussion

Our findings are similar to recent research on implementing evidence-based mental health programs in community settings. Allocating adequate staff resources, training, and communication is essential when integrating a new program into settings that have a high staff turnover and among staff who have a heavy workload and limited time (23,24). Our study highlights the value of considering where evidence-based programs are delivered (16); contextual elements include organizational structure and process, resources, norms and attitudes, and policies and incentives (7,25). Cretin and colleagues (26) note that, particularly for chronic care management programs, effective implementation requires a specified set of activities coordinated at the system, organizational, program, or practitioner level. These activities are often provided by the research team during effectiveness and efficacy studies and should be articulated by each adopting agency to facilitate effective translation.

These findings have informed the development and execution of a strategic implementation plan at ADS to improve PEARLS program delivery. During the past 3 years, ADS has expanded program eligibility criteria, standardized implementation processes, and conducted new education and training activities to address barriers to program implementation and provide education and support that was lost after the efficacy trial ended. ADS has experienced modest improvements in rates of referral (from 9% to 15%) and enrollment (from 4% to 8%) during this time. Findings from this research have also benefited other PEARLS programs around the country, many of which experience similar issues. Study findings are shared during monthly technical assistance calls with PEARLS providers, who are able to adapt and use them to augment program delivery in their agencies.

This study provides a detailed picture of implementation from the perspectives of those involved; contrary to an efficacy study, in which context is capable of being controlled, "implementation research occurs in real world settings distinguished by their complexity and variation in context" (27). Interviews allowed researchers to better understand the historical and implementation climates without preconceived notions of what was or was not working. Agency staff also contributed to improving program implementation, which was noteworthy, given feelings of disengagement among staff in an environment of mounting workloads in a bureaucratic agency.

This study had several limitations. Bias is inherent if participants have preexisting, ongoing relationships or if they feel more or less likely to participate on the basis of perceived incentives or liabilities of participation. In groups, participants may respond according to what other people say (social desirability bias) or not offer new ideas when the group is in agreement ("groupthink" bias) (28). The interviews were organized by agency role to facilitate a safer setting (eg, participants were placed in groups without their supervisors or supervisees, which can create tension or unequal power dynamics). Another limitation was using both one-on-one interviews and focus group interviews to collect data. We used different methods because it was important to keep PEARLS roles separate to gather the richest data possible. We also shared findings with participants and other staff to verify whether results matched perspectives. Although the appropriateness of member checking has been debated (29), our intent was to confirm whether our findings were accurate and not to force them on participants (30).

Findings suggest that purposive sampling and thematic analyses can identify a range of key factors for implementation of evidence-based programs. These factors have helped target improvements in PEARLS delivery at ADS and are also relevant for researchers and practitioners interested in improving translation of evidence-based practices.

## Figures and Tables

**Table.** Characteristics of Participants (N = 38), Qualitative Study on an Evidence-Based Depression Care Management Program (PEARLS), Seattle, Washington, 2008

**Interview or Focus Group**	**No. of Participants**	**Types of Participants**
1	4	Supervisors and administrators not directly involved in program implementation (team supervisors of case managers, educator/trainer)
2	1	Clinical supervisor
3	2	Case managers
4	5	Case managers
5	6	Supervisors and administrators directly involved in program implementation (agency director, case management program directors, data manager, program supervisor)
6	4	Case managers
7	7	Case managers
8	2	Counselors (interventionists)
9	4	Former program participants
10	3	Former program participants
